# Stewardship Behaviour Among Residents of the Great Barrier Reef Region and the Role of Self-Efficacy

**DOI:** 10.1007/s00267-026-02525-x

**Published:** 2026-06-05

**Authors:** Jane Dousset, Matthew I. Curnock, Ju-Han Zoe Wang, Tracy Schultz, Angela J. Dean

**Affiliations:** 1 James Cook University, Townsville, QLD Australia; 2 CSIRO Environment, Townsville, QLD Australia; 3 College of Arts, Society and Education, James Cook University, Cairns, QLD Australia; 4 School of the Environment, The University of Queensland, Brisbane, QLD Australia; 5 Centre for Biodiversity and Conservation Science, The University of Queensland, Brisbane, QLD Australia; 6 School of Agriculture and Food Sustainability, The University of Queensland, Brisbane, QLD Australia

**Keywords:** Great Barrier Reef, self-efficacy, environmental stewardship, reef stewardship, climate change, trust

## Abstract

As the Great Barrier Reef (GBR, the Reef) faces unprecedented threats, reducing anthropogenic pressures and fostering stewardship within coastal communities are among the strategic priorities identified in the Australian and Queensland Government’s Reef 2050 Long-Term Sustainability Plan. While research indicates that Australians have limited awareness of reef stewardship actions, few studies have specifically examined patterns and enablers of stewardship in reef communities. To address this gap, we surveyed residents of the GBR region (*n* = 2317) to examine (i) the types and prevalence of Reef stewardship actions amongst Reef residents (ii) the role of self-efficacy (i.e. an individual’s confidence in their own ability to achieve an outcome) in shaping stewardship behaviour on the GBR and (iii) factors that influence self-efficacy in relation to Reef-protection. We found that many residents report they perform stewardship actions related to pollution, like beach cleanups, with few respondents identifying actions related to climate mitigation. Analysis examining how factors shape stewardship and self-efficacy revealed a complex pattern of findings. On one hand, factors such as feeling a moral obligation to act supported stewardship engagement and self-efficacy. In contrast, being satisfied with reef management was associated with lower rates of stewardship and self-efficacy. These findings suggest a potential trade-off in perceptions of personal and institutional responsibility for action, and highlight the need promote narratives of shared responsibility when promoting stewardship.

## Introduction

Marine ecosystems globally are experiencing increasing pressures. While government and private sector organisations have an important role to play in responding to environmental change, broader community members make an important contribution to environmental protection and restoration (Dean et al. 2025). Such actions can be referred to as ‘environmental stewardship’, which is typically defined as ‘the actions taken by individuals, groups, or networks of actors, with various motivations and levels of capacity, to protect, care for or responsibly use the environment in pursuit of environmental and/or social outcomes in diverse social-ecological contexts’ (Bennett et al. 2018, pg. 599).

There are diverse factors that influence stewardship behaviours, including an individuals’ capacity or motivations, as well as a range of contextual factors (ibid.). Substantial research has explored drivers of many types of environmental stewardship, and a key determinant is an individual’s belief that their actions will result in a desired outcome—‘self-efficacy’. This paper summarises the literature around environmental stewardship and the role of self-efficacy and explores them in the context of a case study of stewardship in Australia’s Great Barrier Reef (GBR; the Reef). Understanding the current state of environmental stewardship by residents in this region, what influences their stewardship, as well as factors that may contribute to higher (or lower) levels of self-efficacy can provide valuable insights for stewardship initiatives. This empirical case study will contribute not only to improving stewardship for the GBR but will also strengthen broad theoretical understandings about factors that support both environmental stewardship and efficacy.

### Frameworks for Understanding Environmental Stewardship and its Drivers

Bennett and colleagues (2018) developed a framework to conceptualise stewardship comprised of six broad elements (context, actors, motivations, capacity, actions and outcomes); plus potential leverage points that could be utilised to achieve desired environmental and social outcomes. Actions are the actual stewardship behaviours whilst outcomes are the results of the actions. Context, actors, motivations and capacity are the overarching elements which influence performance of stewardship actions. Context encompasses factors, such as environmental change in the local setting, as well as social, economic, political and cultural factors (Bennett et al. 2018). For example, this could include concern about the health of the environment and threats to it (Lyon et al. 2018; Ogunbode et al. 2022) or trust in institutions which manage the environment (Moon 2017; Sautkina 2021). Actors comprise characteristics of individuals and groups involved in the stewardship setting and encompasses both demographic factors (Soares et al. 2021) and psychological perceptions of identity, dependence, and attachment to the environment within that setting (Confente and Scarpi 2021). Motivations are related to what inspires action, and stem from both internal beliefs (intrinsic motivations) or external rewards and sanctions (extrinsic motivations) (Bennett et al. 2018). For example, valuing the natural environment (Rees et al. 2015) (i.e. ecosystem services; Gainsburg et al. 2023), emotions such as hope or sadness (Comtesse et al. 2021; Dean and Wilson 2023; Innocenti et al. 2023), and beliefs about the actions or expectations of others (Borg et al. 2020; Johnson et al. 2021) may all motivate stewardship action. Capacity is the final component and comprises individual knowledge, skills, and resources (Martin et al. 2017; Gottwald and Stedman 2020) plus institutional factors such as policies and political structures (Bennett et al. 2018).

While several frameworks and definitions have been proposed to conceptualise environmental stewardship (e.g. Enqvist et al. 2018; Mathevet et al. 2018; Thomas and Romolini 2023; McLeod et al. 2024), our study draws primarily on Bennett et al.'s framework. Bennett et al.'s framework has been widely applied and used in both marine and terrestrial stewardship research and has been specifically adapted for the GBR context to guide stewardship monitoring (Hobman et al. 2025). Importantly, Bennett’s framework also informed the design of the SELTMP (Social and Economic Long-Term Monitoring Program) social survey instrument from which our data were drawn (Curnock et al. 2022), meaning the survey items align closely with the framework’s elements of context, actors, motivations, and capacity.

### The Role of Self-efficacy in Environmental Stewardship

Psychological research posits that feeling able to act is a necessary ingredient to motivate one’s action. This is termed ‘self-efficacy’ and has been defined as the belief ‘in one’s capabilities to organise and execute the courses of action required to manage prospective situations’ (Bandura 1995, pg. 2). Concepts of efficacy are incorporated into many behavioural theories, including Protection Motivation Theory (Rogers 1975), the Theory of Planned Behaviour (Ajzen 1991), and the Norm Activation Model (Schwartz 1977). Aligned with these theories, much empirical research across education, public health, and organisational behaviour emphasises the importance of self-efficacy to behaviour (e.g. Hammond and Feinstein 2005; Bağ and Mollaoğlu 2010; Kim et al. 2024). In relation to environmental behaviours, research demonstrates that self-efficacy promotes diverse actions, including recycling behaviour (Tabernero and Hernández 2011), reduction in residential energy use (Barry et al. 2016), waterway stewardship (Dean et al. 2024) and private land conservation (Uebel et al. 2021; Pradhananga and Davenport 2022). Despite the centrality of self-efficacy in much research about pro-environmental behaviours, it is not an explicit component of Bennett’s stewardship framework, rather, it is regarded among a broader range of intrinsic motivators (p. 602, Bennett et al. 2018).

Self-efficacy has an important influence on behaviour across diverse settings, but at an individual level, self-efficacy can vary between tasks and across different contexts (Schwarzer and Luszczynska 2022). For example, an individual may have high self-efficacy about a certain environmental action, such as recycling, but have lower self-efficacy about another, such as climate mitigation (Whitmarsh et al. 2011). This variability suggests that empirical case studies of self-efficacy (in relation to environmental stewardship) are likely to identify a variety of factors which may enable or hinder self-efficacy. Understanding these local factors can potentially be useful to identify barriers and ‘leverage points’ to facilitate improved uptake and outcomes of stewardship initiatives by promoting greater self-efficacy. In the context of reef stewardship, recent research suggests that self-efficacy is an important driver of personal action (Dean and Wilson 2023), however many community members perceive that they cannot really make a difference, suggesting low efficacy (Waters et al. 2025). This raises the importance of examining not only how self-efficacy influences reef stewardship, but what factors shape self-efficacy for reef stewardship.

### What Factors Contribute to Self-efficacy?

Bandura (1977) argued that four factors support feelings of self-efficacy: (i) past accomplishment (experience of performing the action); (ii) vicarious experience (watching and learning from others performing the action); (iii) verbal persuasion (such as encouragement from others); and (iv) physiological state (such as associating the action with positive emotions). Many of the motivational and capacity factors inherent to an individual are likely to have a mediating effect on their self-efficacy. These include knowledge and experience (Ineson et al. 2013), the actions or expectations of others (social norms) (Cuganesan et al. 2018; Kim et al. 2021), and positive emotions like hope and pride (Capaldi et al. 2014; Bratman et al. 2021). These motivating factors may foster self-efficacy by promoting skill development and confidence. However, the role of contextual and motivational variables on self-efficacy may vary between different cases and settings, as well as between individuals in those settings, which means empirical case studies play an important role in confirming factors important to high self-efficacy in specific contexts. For example, the potential link between negative emotions—such as sadness about environmental degradation or concerns related to environmental threats—and self-efficacy is not clear. While Bandura argues that negative emotions may undermine self-efficacy and serve to demotivate behavioural intent, other research has indicated that negative emotions can also motivate protective actions (Comtesse et al. 2021; Innocenti et al. 2023). While perceptions about severity of environmental problems and need for stewardship may motivate action in many settings; in the context of coral reef degradation and worsening climate impacts, perceptions about the scale of environmental problems may undermine self-efficacy (Dean and Wilson 2023; Said and Wölfl 2025).

### A Case Study of Stewardship and Self-efficacy on The Great Barrier Reef

The Great Barrier Reef is the largest reef system in the world and is a UNESCO World Heritage Area. Presently, the Reef is being threatened by impacts from climate change including rapidly increasing sea temperatures, acidification, and increased risk of severe cyclones (McWhorter et al. 2024). Additional threats that undermine Reef resilience include Crown of Thorns starfish, and pollution from chemicals, sediments, and nutrients (Great Barrier Reef Outlook Report 2024). In response to these threats, the Reef 2050 Long-Term Sustainability Plan (the Plan) (Commonwealth of Australia 2023) was created in 2015 as an overarching strategic policy requiring action from all levels of government. Environmental stewardship is recognised as an important part of Reef management under this Plan, with one objective stating that ‘people and communities [should] take individual and collective action to maintain Reef resilience’ (Commonwealth of Australia 2023, pg. 18). The objective recognises that the ‘community has a role to play in protecting the benefits of the Great Barrier Reef for current and future generations’ (Commonwealth of Australia, 2021, pg. 21). To support stewardship monitoring on the GBR, Hobman et al. (2025) adapted Bennett et al.’s (2018) environmental stewardship framework for the GBR context and identified that key knowledge gaps include not only baseline information on stewardship in Reef communities, but also understanding of the factors that enable stewardship. Our study surveyed residents within the Reef region to address these gaps. Our aims were to:

1. Provide a descriptive snapshot of Reef stewardship behaviours
2. Identify factors that support Reef stewardship
3. Identify factors that promote self-efficacy for Reef stewardship.

## Methodology

### Survey Dataset

We used publicly available survey data of 2317 adult residents within the Great Barrier Reef catchment region, Australia (Curnock et al. 2024). Collected in June and July 2023, the dataset is demographically representative of the regional population, administered by the Commonwealth Scientific and Industrial Research Organisation’s (CSIRO) Social and Economic Long-Term Monitoring Program (SELTMP; Hobman et al. 2024). This data forms part of a time-series for monitoring residents’ uses and values of the GBR. In 2023, survey items about reef stewardship were included as part of a broader stewardship monitoring program.^1^ The dataset and survey questions are publicly available for download (10.25919/v7ff-zb64), and further details on the data collection method and survey design are described by Hobman et al. (2024).

### Patterns of Reef Stewardship Action

Near the beginning of the survey, respondents were asked, ‘Do you currently undertake any actions to help protect the GBR? Please consider a range of things that you may be doing, in a formal group or independently.’ Response options were: (1) ‘Yes, as part of a formal group’, (2) ‘Yes, independently (i.e. not as part of a formal group)’, (3) ‘Yes, a mix of activities with a group and independently’, (4) ‘No’, and (5) ‘Unsure’. Respondents who selected options 1–3 were then asked to: ‘Please describe the types of things that you are doing to help the GBR’ (open ended). For this study, we were interested in if respondents were engaging in stewardship action at all, rather than focusing on if they were doing so as an individual or in a group environment. Therefore, stewardship action was coded as a binary variable (1=yes. 0=no). All ‘yes’ responses were coded as yes, whilst no and unsure responses were classified as no.

Open-ended responses were coded using content analysis (Krippendorf 2018). Drawing on previous research methods and frameworks (Dean et al. 2021; Hobman et al. 2022), we coded responses into four main categories of stewardship action: on-ground actions, social and civic, household and business and non-specific. Our coding process was iterative, involving two researchers who contributed to (i) assignment of codes to the text responses, (ii) review and critique of each other’s codes, and (iii) group deliberation to finalise coding outcomes for ambiguous cases. Multiple codes were assigned to responses in which respondents indicated they participated in different activities (or activity types).

### Self-efficacy for Reef Stewardship

Many approaches to measuring self-efficacy are focused on specific actions (‘task specific’, e.g. feeling able to write a letter to a politician about climate change). However, the large number of stewardship actions make it challenging to quantify task specific self-efficacy for all options. In addition, research indicates low efficacy for reef stewardship relates to the general domain of being able to make a difference for the reef (Waters et al. 2025). Given this, we drew on methods used to assess what is referred to as ‘domain specific’ self-efficacy (Grether et al. 2018; Lu et al. 2023), to focus on feeling able to act in a specific situation or context, in this case Reef protection. Self-efficacy for Reef stewardship was assessed using a single item. Respondents rated their agreement with the statement ‘I feel that I can make a personal difference to the health of the GBR’ (10-point scale, from 1=strongly disagree to 10=strongly agree). This same item was then also used as a response variable to identify what factors may relate to self-efficacy itself.

### Explanatory Factors

Nineteen explanatory variables were chosen for analysis, based on their alignment with framework elements (Bennett et al. 2018) (Table 1). *Context* incorporated perceptions about Reef health, threats, and trust in Reef science and management (Table 1). *Actors* incorporated demographics, place identity, and income associated with the GBR. *Motivations* included self-efficacy, psychological states such as emotions, social norms, and the sense of moral obligation towards and value for the GBR. *Capacity* included factors such as education, income, and knowledge about how to help the Reef (Table 1). These were then assessed against the response variable of Reef stewardship action (Table 1). In the second analysis, self-efficacy then acted as the response variable, so the eighteen remaining explanatory variables (i.e. all bar self-efficacy) were then assessed against self-efficacy. For the explanatory variables where a 5 or 10 point scale was used to capture responses, selecting number 1 meant respondents strongly disagreed with the statement, whilst selecting number 5 or 10 meant respondents strongly agreed.

**Table 1:** Factors explored for their relationship with Reef stewardship and self-efficacy

Element	Variable	Survey question
Response variables	Stewardship action	Do you currently undertake any actions to help protect the GBR? Please consider a range of things that you may be doing, in a formal group or independently.
	Self-efficacy (*also an explanatory variable in the stewardship regression)*	I feel that I can make a personal difference in improving the health of the GBR (10-point scale)
Explanatory variables: context	Management satisfaction	Overall, I feel satisfied with how the GBR is managed (10-point scale)
	Perceived Reef health	I think the Reef and its catchments are currently in good health (10-point scale)
	Perceived threat (climate change)	Rate the extent to which you think climate change represents a threat to the GBR (5-point scale)
	Perceived threat (rubbish)	Rate the extent to which you think rubbish and marine debris represents a threat to the GBR (5-point scale)
	Trust in science	Considering the information you receive about the GBR, how much do you trust the information that comes from the following groups (scientists from research institutions)? (10-point scale)
Explanatory variables: actors	Age	How old were you at your last birthday?
	Gender	How do you describe your gender?
	Place identity	The GBR is part of my identity (10-point scale)
	Reef-related income	What proportion of your household income is from GBR-related businesses or employment (e.g., tourism, restaurants, boating, retail)?
Explanatory variables: motivations	Hope	All things considered, I feel hopeful about the future of the GBR (10-point scale)
	Moral obligation	I feel morally obligated to reduce any impacts I might personally have on the GBR (10-point scale)
	Normative belief	I think that most people in my local area try to reduce any impacts they might have on the GBR (10-point scale)
	Pride	I feel proud the GBR is a World Heritage area (10-point scale)
	Sadness	I feel sad when I think about the outlook for the GBR (10-point scale)
	Value of existence	The fact that the GBR exists even if I don’t use or benefit from it (10-point scale)
Explanatory variables: capacity	Education	Highest level of education completed? (‘school year 10 or below’ to ‘postgraduate degree’)
	Income	Please indicate your approximate total annual income (before tax) for your household (‘$1 to $20,000 per year’ to ‘more than $300,000 per year’)
	Knowledge	I have a good understanding of the things I can do to help the GBR (10-point scale)

### Statistical Analysis

We used logistic regressions as the appropriate tool to test potential relationships between hypothesised explanatory variables and the two response variables (stewardship action and self-efficacy; Table 1). Two regression analyses were conducted: one examining relationships between explanatory variables (including self-efficacy) and the performance of Reef-protecting actions (‘stewardship action’), the other between explanatory variables and self-efficacy. Binary logistic regression was used for the Reef-protecting actions regression as the response variable was dichotomous, whilst ordinal logistic regression was used for the self-efficacy regression as the response variable was on a 10-point Likert scale (McCullagh 1980).

Models were checked to ensure absence of collinearity and adherence to other assumptions (Stoltzfus 2011). Ordinal logistic regression assumes proportional odds (McCullagh 1980). The proportional odds assumption for the ordinal logistic regressions was tested using a Brant test (Brant 1990). The assumption failed for several variables within the ordinal model. To combat this, the data for both the response as well as the explanatory variables were re-grouped for the ordinal regression only. This involved converting data from 5, 6, or 10 categories down to only 3 ‘low’, ‘medium’ and ‘high’ categories. The model was then re-run on these 3 categories, and the Brant test again used to test for proportional odds. For all variables except ‘moral obligation’ the regrouping to 3 categories helped to rectify the violation of proportional odds. It is argued that the assumption of proportional odds can be overstressed, and that observed trends remain useful even if assumptions are violated (Han et al. 2023; Powell et al. 2024). Given this and the knowledge gaps related to self-efficacy, we retained moral obligation in the model, reported on it in this paper. All analyses were performed using R version 4.3.0. Ordinal regressions were run using ‘polr’ function in the ‘MASS’ package (Venables and Ripley 2002). The Brant test was run using the ‘Brant’ package (Schlegel and Steenbergen 2020).

## Results

### Participant Characteristics

Of the 2317 survey respondents, the largest age group (23%) was those between 60-69 years old, following by those between 30 and 39 years old (19%; The least represented group include those between 18 and 29 years old (11%), and those above 75 years old (7%). 45% of respondents identified as female, and most (75%) respondents had completed some form of higher education.

### Residents’ Self-reported Stewardship Actions for the GBR

To categorise self-reported stewardship actions among GBR residents, we analysed the response to the question, ‘please describe the types of things that you are doing to help the GBR’ from those who indicated that they had performed actions to protect the Reef. One third of respondents (*n* = 791, 34%) reported that they performed a Reef stewardship action(s)—82 (3.5%) do so as part of a formal group, 528 (22.8%) reported they do so independently, and 181 (7.8%) reported they do so both as part of a formal group and independently. Overall, 1342 (57.9%) respondents report they take no action whilst 184 (7.9%) were unsure if they perform any actions. Of all survey respondents, 31% (*n* = 719) described the type of stewardship action they perform. The most frequently reported type of action was ‘On-ground actions’ (Table 2), reported by 25.8% of respondents. Within this category, debris removal was the dominant activity (19.9%, *n* = 456), with participants describing various forms of waste collection such as beach cleanups and picking up rubbish. Other actions, such as following fishing rules, participation in citizen science activities, and habitat restoration, were less common. ‘Social and civic’ stewardship actions were the next most frequently reported (8.0%, *n* = 184). Within this category, education and advocacy efforts were most common (5.3%, *n* = 121), with participants engaging in activities such as sharing information on social media and talking to others about reefs and climate change. ‘Household and business-related practices’ were reported by 7.1% (*n* = 163) of participants. Within this category, plastic and waste reduction was most prevalent (4.2%, *n* = 97). Carbon emissions reduction behaviours were reported by just 1.3% respondents.

**Table 2:** Types of Stewardship Actions for the GBR as reported by survey respondents

Type of action	Example responses	%	*n*
**On-ground**		**25.8**	**593**
Debris removal	‘Participating in beach cleanups’ ‘Picking up trash’ ‘Collect any rubbish found floating or washed up on beaches’	19.9	456
Following rules	‘Strictly following fishing/crabbing rules.’ ‘Fishing in designated fishing areas’ ‘No rubbish overboard’	4.3	98
Citizen science	‘Turtle nest monitoring’ ‘Monitoring our local reef’ ‘Eye on the Reef’ app’	2.9	67
Habitat restoration	‘Rehabilitate sand dunes’ ‘Landcare and tree planting’ ‘Coral planting’ ‘Revegetating an eroding creek’ ‘Participate in Coastcare replanting activities’	1.4	32
Other	‘Wildlife rescue’	4.4	102
**Social and civic**		**8.0**	**184**
Education and advocacy	‘Publish a [conservation newsletter] on social media’ ‘Online climate activism, signing petitions’ ‘Involved with a local lobby group’ ‘Talking about reefs and climate change’	5.3	121
Donating money	‘Donate money to [reef conservation] organisation’ ‘Donate to causes to protect reef’	1.8	41
Volunteering for an organisation	‘Member of Local Marine Advisory Committee’ ‘Volunteer with a marine conservation organisation’	1.6	36
Other	‘Report injured wildlife’ ‘Report illegal rec fishing in green zones’	0.7	17
**Household and business**		**7.1**	**163**
Plastic and waste reduction	‘Less plastic use’ ‘Reduce my personal waste’	4.2	97
Land and garden	‘Minimise my use of chemicals’ ‘Careful with what I use in the garden’	2.7	62
Emissions reduction	‘Reduce carbon footprint’ ‘Save more energy’	1.3	30
Other	‘Eat less fish’ ‘Conserving water, Composting’	0.8	18
Non-specific	‘Finding out more’ ‘Looking after the environment’	**1.1**	**25**

### Factors Related to Reef Stewardship Action

#### Context

**Fig. 1:**
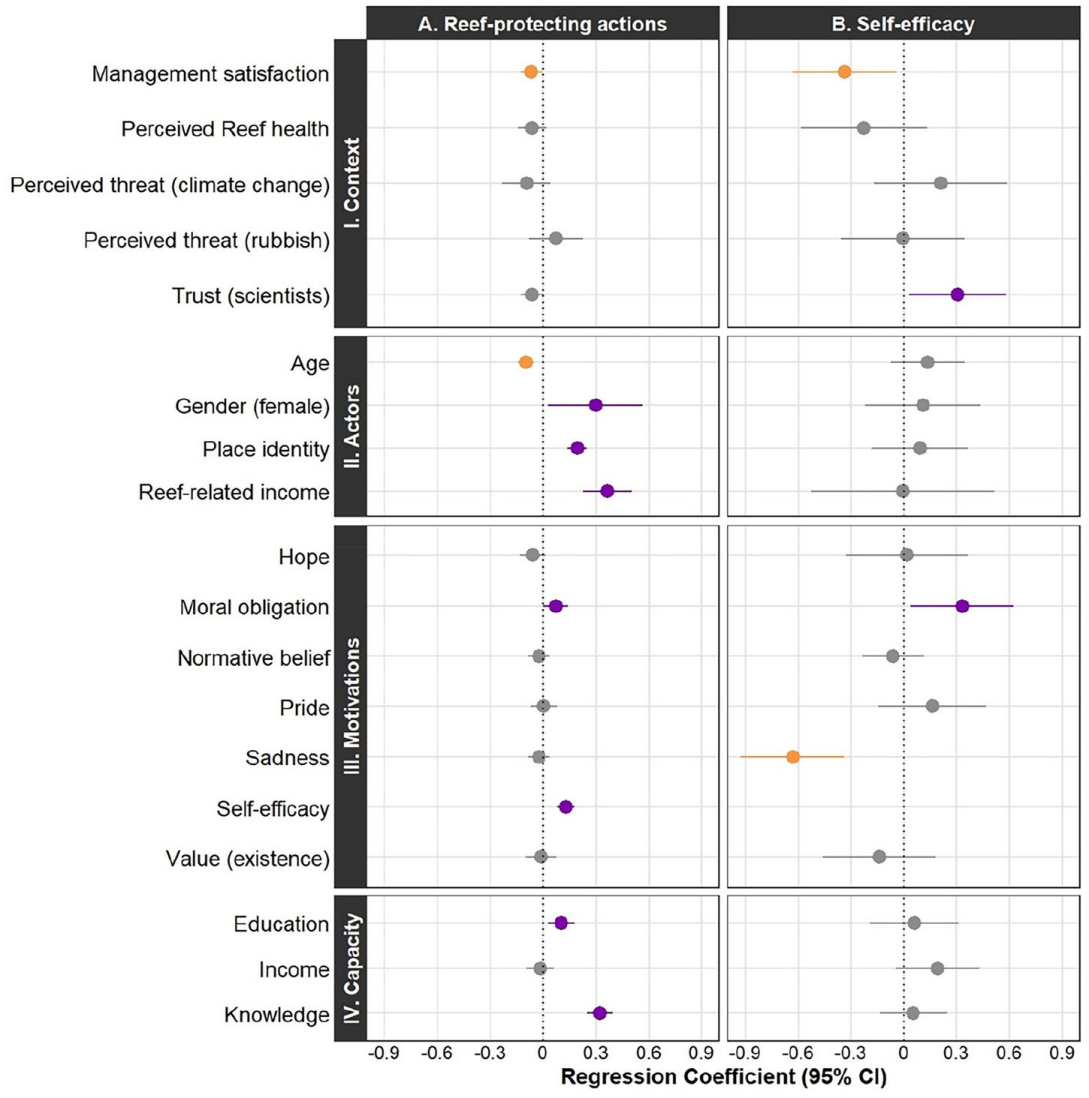
Forest plot showing the outcome of two logistic regressions. Response variables are listed on the top *x-*axis and explanatory variables on the *y-*axis. Explanatory variables are grouped into elements from the environmental stewardship framework (Bennett et al. 2018). The points represent the regression coefficient (b) and the bars either side of each point represent the 95% confidence interval of the coefficient. The colours represent significance: purple for coefficients which are significant in the positive direction, grey for non-significance, and orange for coefficients which are significant in the negative direction. The size of the regression coefficient is indicated by the scale on the bottom *x* axis

Binary logistic regression examined factors associated with self-reported stewardship action. Among all contextual factors, management satisfaction was the only context factor associated with Reef-protecting actions; it had a negative significant relationship to performance of Reef-protecting actions (Fig. 1A-I; Table A.1). This indicates that respondents who reported higher satisfaction with the current management of the Reef were less likely to report engaging in Reef-protecting actions.

#### Actors

A range of actor characteristics were positively associated with greater engagement in stewardship, including younger age, female gender, having a stronger Reef identity, and gaining income from the reef (Fig. 1A-II; Table A.1).

#### Motivation

Self-efficacy exhibited a positive relationship with Reef stewardship, where those who felt more able to make a difference to Reef health were more engaged in stewardship (Fig. 1A-III; Table A.1). When assessing other motivational factors, respondents who reported greater sense of moral obligation were also more likely to engage in stewardship. No associations were observed for other motivational factors. (Fig. 1A-III; Table A.1).

#### Capacity

Both education and knowledge were associated with stewardship (Fig. 1A-IV; Table A.1): respondents reporting higher educational attainment and greater understanding of how to help the Reef were more likely to report performing stewardship actions. No effect of income was observed.

### Factors Related to Self-efficacy

#### Context

Management satisfaction also had a negative significant relationship to self-efficacy (Fig. 1B-I; Table A.1). This means respondents who reported higher satisfaction with the current management of the Reef were more likely to report lower levels of self-efficacy. In contrast, those with higher levels of trust in the scientists and science institutions related to the Reef were more likely to report stronger self-efficacy for Reef stewardship (Fig. 1B-I; Table A.1). Perceptions about threats and Reef health were not associated with self-efficacy.

#### Actors

No actor characteristics (age, gender, place identity, reef-related income) were associated with self-efficacy (Fig. 1B-II; Table A.1).

#### Motivation

Respondents describing a stronger moral obligation to protect the Reef were more likely to report stronger self-efficacy (Fig. 1B-III; Table A.1). Sadness had a negative relationship with self-efficacy (Fig. 1B-III; Table A.1), where respondents who reported more sadness about the Reef were more likely to report lower self-efficacy for Reef stewardship. Hope, normative belief, pride, and value (existence) were not associated with self-efficacy (Fig. 1B-III; Table A.1).

#### Capacity

No capacity variables (education, income, knowledge) exhibited a significant relationship to self-efficacy (Fig. 1B-IV; Table A.1). Note that all regression results can be reviewed in Online Resource 1.

## Discussion

This is the first study to quantify the prevalence and types of stewardship actions taken by residents of the GBR region. These findings show how residents interpret and ‘operationalise’ ‘GBR stewardship’—revealing specific actions they associate with protecting and caring for the Reef. Such self-reported behaviours provide insight into how community members conceptualise their role in Reef protection and what activities they prioritise as stewardship actions. Analysis examining how context, actors, motivations, and capacity factors relate to stewardship and self-efficacy revealed an intricate pattern of findings and highlighted potential leverage points for Reef practitioners. On one hand, factors such as feeling a moral obligation to act supported stewardship engagement and self-efficacy. In contrast, being satisfied with Reef management was associated with lower rates of stewardship and self-efficacy. These findings suggest a complex interaction of variables in settings such as the GBR, where perceptions of institutional responsibilities and personal obligations may play a key role.

### Conceptualisations of Stewardship

‘On-ground’ activities (such as beach clean-ups) and forms of environmental advocacy (such as online petitions) were the most reported among GBR residents. This indicates that rubbish and pollution might be at the forefront of resident’s minds as a predominant issue facing the Reef—a finding consistent with existing research conducted about stewardship for the GBR (Dean et al. 2021), and waterways (Dean et al. 2024). This finding may also highlight that accessible stewardship actions are those most favoured by residents. Given that climate change poses the greatest threat to the Reef (Great Barrier Reef Outlook Report 2024), this focus on litter and debris potentially distracts from an important opportunity for strengthening community engagement in climate actions. It is notable that climate change risk perceptions among GBR residents have fluctuated over the last decade, with a trend of growing climate scepticism since 2017 (Hobman et al. 2024). However, most residents in the GBR region still recognise climate change as ‘a threat to the GBR requiring immediate action’ (ibid.), so perhaps in situ actions such as collecting rubbish may represent an ‘easier’ or more accessible action than tackling ‘global’ issues such as climate change (Said and Wölfl, 2025) so are therefore more often performed by residents.

‘Social and civic’ actions were the second most reported type of stewardship. This aligns with research indicating that advocacy and activism—such as attending climate strikes, writing to politicians, or signing petitions—have become increasingly popular among younger generations (Pickard 2019, 2022). Within this and ‘household actions’, some reported actions did specify focusing on responding to climate change. The diverse stewardship actions described are likely to exhibit diverse types of impact. Given this, there remains significant scope to better understand how different actions contribute to better outcomes for the reef and strengthen community engagement in high impact actions (Hofman et al. 2020).

### Self-efficacy and Moral Obligation

Our finding that higher levels of self-efficacy were positively associated with Reef stewardship confirms established associations between self-efficacy and stewardship behaviour in the literature (as discussed in section ‘Introduction’), particularly in the context of the GBR (Tabernero and Hernández 2011; Barry et al. 2016; p. 602, Bennett et al. 2018; Uebel et al. 2021; Pradhananga and Davenport, 2022; Dean et al. 2024). Similarly, stronger moral obligation to reduce personal impact on the Reef was related to greater likelihood of performing Reef-protection actions and greater self-efficacy. This observation aligns with several behaviour theories discussed earlier (Schwartz 1977; Ajzen 1991), which all suggest that morals are a strong driver of behaviour. This may be because living up to these obligations and values may help an individual avoid feelings of guilt, distress or responsibility for a degrading environment (Eden, 1993; Rees et al. 2015; Schwartz, 1977). Our finding is consistent with other research in diverse social groups, including of farmers (Pradhananga and Davenport 2022), fishers (Ngoc et al. 2024), and national park visitors (Wu et al. 2021).

### Perceptions of Management and Relationship with Stewardship

The finding that Reef residents who reported greater satisfaction with the management of the Reef were less likely to participate in stewardship actions and reported lower self-efficacy is intriguing. Research indicates a complex interplay between an individuals’ perception of management agencies, the role of science, and personal stewardship action and self-efficacy. For example, a study on environmental stewardship behaviours, found that greater institutional trust was associated with less willingness to adopt ‘lifestyle’, ‘on-ground’ or ‘citizenship’ environmental stewardship behaviours (Church et al. 2023). Our findings may be consistent with the above study, suggesting that for some individuals, having trust in or support for Reef management agencies may lead to a sense of complacency, meaning high institutional trust can inadvertently diminish public support for collective environmental action. However, perhaps another suggestion may be that the negative relationship between the two variables is influenced by those respondents who, with greater self-efficacy and a higher propensity for stewardship, have a greater awareness of Reef protection and management issues, and consequently a more critical view of Reef management. Further studies may be required to determine whether this is the case and thus help to determine a leverage point for Reef managers to promote stewardship action by methods such as combating complacency or promoting a better relationship between managers and residents.

The positive association between trust in science and self-efficacy suggests the influence of institutional trust on stewardship may depend on the perceived role of the institution. For example, another study conducted in the GBR found that trust in climate information provided by government agencies was associated with greater stewardship intentions (Wynveen and Sutton 2015). Within this context, trust in an agency that provides information about threats to natural areas may inspire action, whereas trust in an agency whose role is to manage natural areas may diminish the perceived need for action. Such trade-offs have been reported in previous research (Lindsay et al. 2017) and suggest the potential for management agencies to emphasise partnerships and shared responsibility when working with communities. We also highlight that overall trust in science related to the Reef has declined among GBR residents since 2017 (Hobman et al. 2022; Curnock et al. 2024), demonstrating the need to strengthen trust amongst GBR residents into the future.

### Negative Emotions May Undermine Self-efficacy

Respondents who felt a greater sense of sadness about the future of the Reef were more likely to have lower self-efficacy. While this aligns with theorisation about the role of positive emotions in supporting self-efficacy (i.e. a positive emotional and physiological state promotes increased self-efficacy) (Bandura, 1977), it does also contrast with several studies which have indicated that negative emotions associated with nature (such as ecological grief) can inspire protective action (Dean et al. 2018; Massingham et al. 2019). Similarly, an experimental study examining approaches to communicating about climate change impacts to the GBR found that negative emotions were an important ingredient for message effectiveness (Waters et al. 2024). Our finding indicates that the influence of negative emotions may depend on the context in which such emotions emerge. Among these cited studies reporting positive associations between sadness and action (Dean et al. 2018; Massingham et al. 2019; Waters et al. 2024), such emotions emerge in response to a novel situation (such as information, nature experience), that may then trigger a new perspective about the need for action. In contrast, our findings that sadness was associated with reduced self-efficacy suggests a different effect. It is possible that within communities living near the Reef, sadness may reflect an ongoing emotional state, that hinders or reduces an individual’s confidence about making a difference. This has been described in the literature as ‘Reef grief’ (Marshall et al. 2019) or ‘eco-paralysis’ (Innocenti et al. 2023) where a sense of hopelessness about the Reef’s future is likely to undermine self-efficacy. This finding perhaps suggests that increased support for residents experiencing ‘Reef grief’ may help to promote self-efficacy for stewardship action.

### Resident Characteristics Shape Stewardship But Not Self-efficacy

Within Bennett’s stewardship framework, our indicators of both actors and capacity incorporated demographic characteristics such as age, gender, and education, as well as Reef-specific factors such as knowledge, identity, and Reef-dependent income. Many of these characteristics were associated with stewardship action, in ways that are well-documented in the literature. For example, research shows that stewardship is higher in younger generations (Prayag et al. 2022; Gomes et al. 2023), women (Brécard et al. 2009; De Silva and Pownall 2014) and those with greater education (Brécard et al. 2009), and procedural knowledge (Dean et al. 2018). The findings about identity and dependence, are supported by place attachment theory, which argues when an individual is attached to a place or dependent on a place, this then leads to greater actions to protect the place (Lewicka 2011). Whilst this study has identified this link exists in the context of the GBR, the relationship between place attachment and stewardship behaviour has similarly been found in a broad range of contexts. For example, in residents’ river preservation in Germany (Gottwald and Stedman 2020), tourists and residents’ awareness of the negative consequences of tourism in Venice (Confente and Scarpi 2021), amongst scuba divers in Taiwan (Kuo et al. 2021), and land preservation in Massachusetts (Lokocz, Ryan and Sadler 2011).

While previous research has suggested that the elements of self-efficacy (Bandura 1977) share common social and psychological foundations with the factors proposed to drive stewardship behaviour and as such, one might expect the factors analysed in this research to be associated with both increased self-efficacy as well as residents’ reported stewardship actions. However, in our analysis, capacity variables (education, income, knowledge) and actor variables (age, gender, place identity, Reef-related income) are not statistically significant predictors of self-efficacy. Previous research has demonstrated that adult-learning is important for increasing self-efficacy (Hammond and Feinstein 2005) as does education in general (Bağ and Mollaoğlu 2010), but potentially these factors are not as important to self-efficacy for stewardship action, or specifically in the context of the GBR. In the context of large-scale environmental issues like Great Barrier Reef degradation, general education may not directly translate into environmental self-efficacy. A person may be highly educated but still feel helpless against macro-level threats like climate change. Further, the lack of association between Reef-specific characteristics, such as place identity and income dependence emphasises that instrumental or psychological connections to ecosystems may not automatically confer a sense of confidence in taking up stewardship actions for these ecosystems. Overall, our results suggest that self-efficacy in this context is driven more by intrinsic, normative factors (moral obligation) and the cognitive framing of the issue (trusting the scientific community that provides actionable solutions), rather than formal education or socioeconomic status.

### Limitations and Further Research

Our empirical case study in the GBR region demonstrates that the contextual, motivational, and capacity factors identified in the environmental stewardship framework (Bennett et al. 2018) can vary between settings—factors identified as being related to self-efficacy and stewardship in the literature did not necessarily apply in the context of the GBR. Further research into other factors assessed in our study as well as drivers of stewardship outlined in other stewardship frameworks may contribute to a more comprehensive understanding of how these factors vary within and between settings, and how they interact to influence engagement in stewardship behaviours. It would be especially interesting to further investigate actor and capacity factors in relation to self-efficacy considering their association with stewardship action, but lack of association with self-efficacy for such actions. Potentially these types of factors are simply not important to self-efficacy for environmental stewardship action, or perhaps there are other types of actor/capacity factors which may be more important in the context of the GBR.

Future research may benefit from the use of more sophisticated statistical analyses of pathways and mediating factors (e.g. by using structural equation modelling). Such studies might help to improve our understanding of non-linear and indirect relationships, especially in relation to self-efficacy and actor or capacity factors.

A limitation common to self-administered surveys about pro-environmental behaviour is their susceptibility to social desirability bias. Social desirability bias is where respondents may over-report or answer in an exaggerated way that makes their responses appear ‘better’ (i.e. more socially desirable) (Edwards 1957). This may have led to self-reports about the stewardship action undertaken by respondents being inflated. We also note that the survey results captured and used in this study represent a snapshot of GBR residents’ stewardship action and self-efficacy at a specific time. As pressures on the Reef change over time, residents’ understanding of protective actions may too evolve, underscoring the importance of monitoring to inform efforts to leverage stewardship outcomes into the future.

## Conclusion

Reef stewardship is a critical component of the sustainable management and protection of the GBR. A key finding of this study is that despite climate change being a major threat to the Reef, many residents are not associating climate mitigation actions with Reef stewardship actions, highlighting a critical disconnect (Spence et al. 2012). Currently, promoting stewardship action is a goal identified in the Reef 2050 Long-Term Sustainability Plan, with the level and drivers of stewardship being called out as poorly understood.

This paper highlights several potential leverage points stewardship practitioners may consider exploiting to promote stewardship and self-efficacy. First, continued communication around climate mitigation can be strengthened by explicitly localising climate threats - framing them into targeted, accessible, community-level actions to overcome psychological distance (Jones et al. 2016; Loy and Spence 2020; Waters et al. 2024). Second, rebuilding of institutional trust may require management agencies to emphasise shared responsibility rather than relying on passive trust. Finally, the provision of increased support for residents experiencing ‘Reef grief’ (Curnock et al. 2019; Marshall et al. 2019) is essential to build self-efficacity required for action. Providing avenues for collective action can help transition residents from passive grief to active, confident stewardship (Walpole and Hadwen 2022). Some examples considering the above three points may include adoption of deliberative democracy models for conservation strategies (Ranger et al. 2016), provision of structured, accessible pathways for citizen science (von Gönner et al. 2023) and community co-management (Ayers et al. 2026).

Additional leverage points may be uncovered through future research using different modelling techniques, to better understanding actor and capacity factors related to self-efficacy. We hope this paper contributes to an improved knowledge of factors associated with stewardship action in the local setting (i.e. for practitioners of GBR stewardship programmes). More broadly, this research serves as a useful case study for the advancement of environmental stewardship theory, offering actionable insights for managing threatened ecosystems globally.

## Supplementary Information

Supplementary information

Supplementary information

## Data Availability

CSIRO SELTMP data and survey questions used in this study are publicly available for download - 10.25919/v7ff-zb64.

## Footnotes

1 People and Reef Organisations Tackling Environmental Change Together (PROTECT), www.protect.qut.protect.qut.edu.au/

## References

[CR1] Ajzen I (1991) The theory of planned behavior. Organ Behav Hum Decis Process 50(2):179–211. 10.1016/0749-5978(91)90020-T.

[CR2] Ayers AL, Kittinger JN, Datta A (2026) Many paths, one destination: assessing community options for co-management of marine resources. Ecol Soc 31(2). 10.5751/ES-16970-310219

[CR3] Bağ E, Mollaoğlu M (2010) The evaluation of self-care and self-efficacy in patients undergoing hemodialysis. J Eval Clin Pract 16(3):605–610. 10.1111/j.1365-2753.2009.01214.x. 19874435 10.1111/j.1365-2753.2009.01214.x

[CR4] Bandura A (1995) Exercise of personal and collective efficacy in changing societies. In: Bandura A (ed.) Self-efficacy in changing societies. Cambridge University Press, p 1–45 10.1017/CBO9780511527692.003

[CR5] Bandura A (1977) Self-efficacy: toward a unifying theory of behavioral change. Psychol Rev 84(2):191–215. 10.1037/0033-295X.84.2.191. 847061 10.1037//0033-295x.84.2.191

[CR6] Barry NA, Harper CM, Berryman C, Farley C (2016) Role of self-efficacy in reducing residential energy usage. J Archit Eng 22(1). 10.1061/(ASCE)AE.1943-5568.0000196.

[CR7] Bennett NJ, Whitty TS, Finkbeiner E, Pittman J, Bassett H, Gelcich S, Allison EH (2018) Environmental Stewardship: a conceptual review and analytical framework. Environ Manag 61(4):597–614. 10.1007/s00267-017-0993-2. 10.1007/s00267-017-0993-2 PMC5849669 29387947

[CR8] Borg K, Curtis J, Lindsay J (2020) Social norms and plastic avoidance: testing the theory of normative social behaviour on an environmental behaviour. J Consum Behav 19(6):594–607. 10.1002/cb.1842.

[CR9] Brant R (1990) Assessing proportionality in the proportional odds model for ordinal logistic regression. Biometrics 46(4):1171. 10.2307/2532457. 2085632

[CR10] Bratman GN, Olvera-Alvarez HA, Gross JJ (2021) The affective benefits of nature exposure. Soc Personal Psychol Compass 15(8):e12630. 10.1111/spc3.12630.

[CR11] Brécard D, Hlaimi B, Lucas S, Perraudeau Y, Salladarré F (2009) Determinants of demand for green products: an application to eco-label demand for fish in Europe. Ecol Econ 69(1):115–125. 10.1016/j.ecolecon.2009.07.017.

[CR12] Capaldi CA, Dopko RL, Zelenski JM (2014) The relationship between nature connectedness and happiness: a meta-analysis. Front Psychol 5:976. 10.3389/fpsyg.2014.00976. 25249992 10.3389/fpsyg.2014.00976 PMC4157607

[CR13] Church EK, Wilson KA, Dean AJ (2023) Broadening our understanding of what drives stewardship engagement: relationships between social capital and willingness to engage in nature stewardship. J Environ Manag 342:118128. 10.1016/j.jenvman.2023.118128. 10.1016/j.jenvman.2023.118128 37210815

[CR14] Commonwealth of Australia (2021) Reef 2050 objectives and goals 2021-2025.

[CR15] Commonwealth of Australia (2023) Reef 2050 long-term sustainability plan 2021-2025.

[CR16] Comtesse H, Ertl V, Hengst SMC, Rosner R, Smid GE (2021) Ecological grief as a response to environmental change: a mental health risk or functional response? Int J Environ Res Public Health 18(2):734. 10.3390/ijerph18020734. 33467018 10.3390/ijerph18020734 PMC7830022

[CR17] Confente I, Scarpi D (2021) Achieving environmentally responsible behavior for tourists and residents: a norm activation theory perspective. J Travel Res 60(6):1196–1212. 10.1177/0047287520938875/FORMAT/EPUB.

[CR18] Cuganesan S, Steele C, Hart A (2018) How senior management and workplace norms influence information security attitudes and self-efficacy. Behav Inf Technol 37(1):50–65. 10.1080/0144929X.2017.1397193.

[CR19] Curnock MI, Marshall NA, Thiault L, Heron SF, Hoey J, Williams G, Taylor B, Pert PL, Goldberg J (2019) Shifts in tourists’ sentiments and climate risk perceptions following mass coral bleaching of the Great Barrier Reef. Nat Clim Change 9:535–541. 10.1038/s41558-019-0504-y.

[CR20] Curnock MI, Nembhard D, Smith R, Sambrook K, Hobman EV, Mankad A, Pert PL, Chamberland E (2024) Finding common ground: understanding and engaging with science mistrust in the Great barrier reef region. PLOS ONE 19(8):e0308252. 10.1371/JOURNAL.PONE.0308252. 39150962 10.1371/journal.pone.0308252 PMC11329155

[CR21] Curnock MI, Pert PL, Maharjan D, Gordon B, Kaniewska P (2022) Design and implementation of social surveys for regional report cards in the Great Barrier Reef catchment. CSIRO Land and Water, Townsville [online]. ISBN: 978-1-4863-1748-6.

[CR22] Dean AJ, Church EK, Loder J, Fielding KS, Wilson KA (2018) How do marine and coastal citizen science experiences foster environmental engagement? J Environ Manag 213:409–416. 10.1016/j.jenvman.2018.02.080. 10.1016/j.jenvman.2018.02.080 29505996

[CR23] Dean AJ, Fielding KS, Smith LDG, Church EK, Wilson KA (2025) Eliciting diverse perspectives to prioritize community actions for biodiversity conservation. Conserv Biol. 39(2). e14372 10.1111/cobi.14372. 10.1111/cobi.14372 PMC11959327 39268844

[CR24] Dean AJ, Gulliver RE, Wilson KA (2021) ‘Taking action for the Reef?’–Australians do not connect Reef conservation with individual climate-related actions. Conserv Lett 14(2). 10.1111/conl.12765.

[CR25] Dean AJ, Uebel K, Schultz T, Fielding KS, Saeck E, Ross H, Martin V (2024) Community stewardship to protect coastal and freshwater ecosystems–pathways between recreation and stewardship intentions. People Nat 6:1452–1468. 10.1002/pan3.10658.

[CR26] Dean AJ, Wilson, KA (2023) Relationships between hope, optimism, and conservation engagement. Conserv Biol 37(2). 10.1111/cobi.14020. 10.1111/cobi.14020 36285591

[CR27] De Silva DG, Pownall RAJ (2014) Going green: does it depend on education, gender or income? Appl Econ 46(5):573–586. 10.1080/00036846.2013.857003.

[CR28] Eden SE (1993) Individual environmental responsibility and its role in public environmentalism. Environ Plan 25:1743–1758. 10.1068/a251743.

[CR29] Edwards A (1957) The social desirability variable in personality assessment and research. Dryden Press.

[CR30] Enqvist JP, West S, Masterson VA, Haider LJ, Svedin U, Tengö M (2018) Stewardship as a boundary object for sustainability research: linking care, knowledge and agency. Landsc Urban Plan 179:17–37. 10.1016/j.landurbplan.2018.07.005.

[CR31] Gainsburg I, Roy S, Cunningham JL (2023) An examination of how six reasons for valuing nature are endorsed and associated with pro-environmental behavior across 12 countries. Sci Rep 13(1):8484. 10.1038/s41598-023-34338-x. 37230999 10.1038/s41598-023-34338-x PMC10209929

[CR32] Gomes S, Lopes JM, Nogueira S (2023) Willingness to pay more for green products: a critical challenge for Gen Z. J Clean Prod 390: 136092. 10.1016/j.jclepro.2023.136092.

[CR33] Gottwald S, Stedman RC (2020) Preserving ones meaningful place or not? Understanding environmental stewardship behaviour in river landscapes. Landsc Urban Plan 198: 103778. 10.1016/j.landurbplan.2020.103778.

[CR34] Great Barrier Reef Marine Park Authority (2024) Outlook Report 2024. https://hdl.handle.net/11017/4069.

[CR35] Grether T, Sowislo JF, Wiese BS (2018) Top-down or bottom-up? Prospective relations between general and domain-specific self-efficacy beliefs during a work-family transition. Personal Individ Differ 121:131–139. https://psycnet.apa.org/doi/10.1016/j.paid.2017.09.021.

[CR36] Hammond C, Feinstein L (2005) The effects of adult learning on self-efficacy. Lond Rev Educ 3(3):265–287. 10.1080/14748460500372754.

[CR37] Han JH, Koch E, Jeffery AD, Reese TJ, Dorn C, Pugh S, Rubenstein M, Wilson JE, Campbell C, Ward MJ (2023) The effect of telemental versus in-person mental health consults in the emergency department on 30-day utilization and processes of care. Acad Emerg Med 30(4):262–269. 10.1111/acem.14688. 36762876 10.1111/acem.14688 PMC11106754

[CR38] Hobman EV, Dyer M, Stone-Jovicich S, Newlands M, Schultz T, Maclean K, Dean AJ (2025) Understanding and monitoring Reef stewardship: a conceptual framework and approach for the Great Barrier Reef. Australas J Environ Manag 32(1):65–85. 10.1080/14486563.2024.2439839.

[CR39] Hobman EV, Mankad A, Pert PL, Chamberland E, Curnock M (2024) Monitoring social and economic indicators among residents of the Great Barrier Reef region in 2023: a report from the Social and Economic Long-term Monitoring Program (SELTMP) for the Great Barrier Reef. CSIRO Environment, Australia. 10.25919/x5ck-4e42

[CR40] Hobman EV, Mankad A, Pert PL, van Putten I, Fleming-Munoz DA, Curnock M(2022) Monitoring social and economic indicators among residents of the Great Barrier Reef region in 2021: a report from the Social and Economic Long-term Monitoring Program (SELMTP) for the Great Barrier Reef. http://hdl.handle.net/102.100.100/600052?index=1

[CR41] Hofman K, Hughes K, Walters G (2020) Effective conservation behaviours for protecting marine environments: the views of the experts. J Sustain Tour 28:1460–1478. 10.1080/09669582.2020.1741597.

[CR42] Ineson EM, Jung T, Hains C, Kim M (2013) The influence of prior subject knowledge, prior ability and work experience on self-efficacy. J Hosp Leis Sport Tour Educ 12(1):59–69. 10.1016/j.jhlste.2012.11.002.

[CR43] Innocenti M, Santarelli G, Lombardi GS, Ciabini L, Zjalic D, Di Russo M, Cadeddu C (2023) How can climate change anxiety induce both pro-environmental behaviours and eco-paralysis? The mediating role of general self-efficacy. Int J Environ Res Public Health 20(4):3085. 10.3390/ijerph20043085. 36833780 10.3390/ijerph20043085 PMC9960236

[CR44] Johnson DN, Shipley NJ, van Riper CJ, Kyle GT, Wallen KE, Landon A, Absher J (2021) Place-based motivations and normative beliefs predict pro-environmental behavior across involvement profiles. J Outdoor Recreat Tour 35: 100377. 10.1016/j.jort.2021.100377.

[CR45] Jones C, Hine DW, Marks ADG (2016) The future is now: reducing psychological distance to increase public engagement with climate change. Risk Anal 37(2):331–341. 10.1111/risa.12601. 26989845 10.1111/risa.12601

[CR46] Kim J, Eys M, Robertson-Wilson J (2021) If they do it, so can I’: a test of a moderated serial mediation model of descriptive norms, self-efficacy, and perceived similarity for predicting physical activity. Psychol Health 36(6):701–718. 10.1080/08870446.2020.1789641. 32620058 10.1080/08870446.2020.1789641

[CR47] Kim BJ, Kim MJ, Lee J (2024) Examining the impact of work overload on cybersecurity behavior: highlighting self-efficacy in the realm of artificial intelligence. Curr Psychol 43:17146–17162. 10.1007/s12144-024-05692-4.

[CR48] Kuo HM, Su JY, Wang CH, Kiatsakared P, Chen KY (2021) Place attachment and environmentally responsible behavior: the mediating role of destination psychological ownership. Sustainability 13(12):6809. 10.3390/SU13126809.

[CR49] Krippendorff K (2018) Content analysis: an introduction to its methodology (4th ed.). SAGE Publications.

[CR50] Lewicka M (2011) Place attachment: how far have we come in the last 40 years?. J Environ Psychol 31(3):207–230. 10.1016/j.jenvp.2010.10.001.

[CR51] Lindsay J, Dean AJ, Supski S (2017) Responding to the Millennium drought: comparing domestic water cultures in three Australian cities. Reg Environ Change 17:565–577. 10.1007/s10113-016-1048-6.

[CR52] Lokocz E, Ryan RL, Sadler AJ (2011) Motivations for land protection and stewardship: exploring place attachment and rural landscape character in Massachusetts. Landsc Urban Plan 99(2):65–76. 10.1016/j.landurbplan.2010.08.015.

[CR53] Loy LS, Spence A (2020) Reducing, and bridging, the psychological distance of climate change. J Environ Psychol 67. 10.1016/j.jenvp.2020.101388

[CR54] Lu H, Chen X, Qi C (2023) Which is more predictive: domain- or task-specific self-efficacy in teaching and outcomes? Br J Educ Psychol 93:283–298. 10.1111/bjep.12554. 36196764 10.1111/bjep.12554

[CR55] Lyon SF, Bidwell D, Pollnac RB (2018) Factors influencing environmentally responsible behavior among coastal recreationists. Coast Manag 46(5):488–509. 10.1080/08920753.2018.1498714.

[CR56] Marshall N, Adger WN, Benham C, Brown K, Curnock MI, Gurney GG, Marshall P, Pert PL, Thiault L (2019) Reef grief: investigating the relationship between place meanings and place change on the great Barrier Reef, Australia. Sustain Sci 14(3):579–587. 10.1007/s11625-019-00666-z.

[CR57] Martin VY, Weiler B, Reis A, Dimmock K, Scherrer P (2017) Doing the right thing’: how social science can help foster pro-environmental behaviour change in marine protected areas. Mar Policy 81:236–246. 10.1016/j.marpol.2017.04.001.

[CR58] Massingham E, Fuller RA, Dean AJ (2019) Pathways between contrasting ecotourism experiences and conservation engagement. Biodivers Conserv 28:827–845. 10.1007/s10531-018-01694-4.

[CR59] Mathevet R, Bousquet F, Raymond CM (2018) The concept of stewardship in sustainability science and conservation biology. Biol Conserv 217:363–370. 10.1016/J.BIOCON.2017.10.015.

[CR60] McCullagh P (1980) Regression Models for Ordinal Data. J R Stat Soc Ser B 42(2):109–127. 10.1111/j.2517-6161.1980.tb01109.x.

[CR61] McLeod LJ, Kitson JC, Dorner Z, Tassell-Matamua NA, Stahlmann-Brown P, Milfont TL, Hine DW (2024) Environmental stewardship: a systematic scoping review. PLOS ONE 9(5):e0284255. 10.1371/journal.pone.0284255 PMC11075856 38713707

[CR62] McWhorter JK, Halloran PR, Roff G, Mumby PJ (2024) Climate change impacts on mesophotic regions of the Great Barrier Reef. Proc Natl Acad Sci USA 121(16). 10.1073/pnas.2303336121 10.1073/pnas.2303336121 PMC11032494 38588432

[CR63] Moon S (2017) The influence of trust on environmental behavior: evidence from South Korea. Int Rev Public Adm 22(2):123–137. 10.1080/12294659.2017.1315232.

[CR64] Ngoc QTK, Xuan BB, Börger T, Hien TT, Van Hao T, Trinh DT, Nghiep VK (2024) Exploring fishers’ pro-environmental behavioral intention and support for policies to combat marine litter in Vietnam. Mar Pollut Bull 200: 116143. 10.1016/j.marpolbul.2024.116143. 38354593 10.1016/j.marpolbul.2024.116143

[CR65] Ogunbode CA, Doran R, Hanss D, Ojala M, Salmela-Aro K, van den Broek KL, Bhullar N, Aquino SD, Marot T, Schermer JA, Wlodarczyk A, Lu S, Jiang F, Maran DA, Yadav R, Ardi R, Chegeni R, Ghanbarian E, Zand S, Karasu M (2022) Climate anxiety, wellbeing and pro-environmental action: correlates of negative emotional responses to climate change in 32 countries. J Environ Psychol 84: 101887. 10.1016/J.JENVP.2022.101887.

[CR66] Pickard S (2019) Young people and DIO politics: do-it-ourselves political participation. In: Politics, protest and young people. Palgrave Macmillan, London. 10.1057/978-1-137-57788-7_12

[CR67] Pickard S (2022) Young environmental activists and Do-It-Ourselves (DIO) politics: collective engagement, generational agency, efficacy, belonging and hope. J Youth Stud 25(6):730–750. 10.1080/13676261.2022.2046258.

[CR68] Powell WR, Vilen L, Zuelsdorff M, Goutman SA, Salamat S, Rissman RA, Bendlin BB, Kind AJH (2024) Association between military service and Alzheimer’s disease neuropathology at autopsy. Alzheimers Dement 20(2):1468–1474. 10.1002/alz.13520. 37965965 10.1002/alz.13520 PMC10917028

[CR69] Pradhananga AK, Davenport MA (2022) I believe i can and should: self-efficacy, normative beliefs and conservation behavior. J Contemp Water Res Educ 175(1):15–32. 10.1111/j.1936-704X.2021.3370.x.

[CR70] Prayag G, Aquino RS, Hall CM, Chen N (Chris), Fieger P. (2022). Is Gen Z really that different? Environmental attitudes, travel behaviours and sustainability practices of international tourists to Canterbury, New Zealand. J Sustain Tour 1–22. 10.1080/09669582.2022.2131795

[CR71] Ranger S, Kenter JO, Bryce R, Cumming G, Dapling T, Lawes E, Richardson PB (2016) Forming shared values in conservation management: an interpretive-deliberative-democratic approach to including community voices. Ecosyst Serv 21(B):344–357. 10.1016/j.ecoser.2016.09.016.

[CR72] Rees JH, Klug S, Bamberg S (2015) Guilty conscience: motivating pro-environmental behavior by inducing negative moral emotions. Clim Change 130(3):439–452. 10.1007/s10584-014-1278-x.

[CR73] Rogers RW (1975) A protection motivation theory of fear appeals and attitude change1. J Psychol 91(1):93–114. 10.1080/00223980.1975.9915803. 28136248 10.1080/00223980.1975.9915803

[CR74] Said N, Wölfl V (2025) Impact of constructive narratives about climate change on learned helplessness and motivation to engage in climate action. Environ Behav 57(1-2):75–117. 10.1177/00139165251315576.

[CR75] Sautkina E, Agissova F, Ivanova A, Ivande K, Kabanova V, Patrakova N (2021) Political, environmental and social determinants of pro-environmental behaviour in Russia. SSRN Electron J. 10.2139/ssrn.3995972

[CR76] Schlegel B, Steenbergen M (2020) brant: Test for parallel regression assumption (0.3-0).

[CR77] Schwartz SH (1977) Normative Influences on Altruism, pp. 221–279. 10.1016/S0065-2601(08)60358-5

[CR78] Schwarzer R, Luszczynska A (2022) Self-efficacy. In: Ruch W, Bakker AB, Tay L, Gander F, (eds) Handbook of positive psychology assessment, Hogrefe Publishing, Göttingen, Germany, p 207–217

[CR79] Soares J, Miguel I, Venâncio C, Lopes I, Oliveira M (2021) Public views on plastic pollution: Knowledge, perceived impacts, and pro-environmental behaviours. J Hazard Mater 412: 125227. 10.1016/J.JHAZMAT.2021.125227. 33951864 10.1016/j.jhazmat.2021.125227

[CR80] Spence A, Poortinga W, Pidgeon N (2012) The psychological distance of climate change. Risk Anal 32(6):957–972. 21992607 10.1111/j.1539-6924.2011.01695.x

[CR81] Stoltzfus JC (2011) Logistic regression: a brief primer. Acad Emerg Med 18(10):1099–1104. 10.1111/j.1553-2712.2011.01185.x. 21996075 10.1111/j.1553-2712.2011.01185.x

[CR82] Thomas AS, Romolini M (2023) Expanding current definitions of environmental stewardship through organizational mission statement analysis. Ambio 52(6):1137–1150. 10.1007/s13280-023-01839-y. 36870032 10.1007/s13280-023-01839-y PMC10160272

[CR83] Tabernero C, Hernández B (2011) Self-efficacy and intrinsic motivation guiding environmental behavior. Environ Behav 43(5):658–675. 10.1177/0013916510379759.

[CR84] Uebel K, Rhodes J, Wilson KA, Dean AJ (2021) Environmental management in the peri-urban region: psychological and contextual factors influencing private land conservation actions. Environ Manag 68:184–197. 10.1007/s00267-021-01487-6. 10.1007/s00267-021-01487-6 34125266

[CR85] Venables W, Ripley B (2002) Modern applied statistics with S (Fourth Edition). Springer. https://www.stats.ox.ac.uk/pub/MASS4/

[CR86] von Gönner J, Herrmann TM, Bruckermann T et al. (2023) Citizen science’s transformative impact on science, citizen empowerment and socio-political processes. Socio-Ecol Pract Res 5:11–33. 10.1007/s42532-022-00136-4.

[CR87] Walpole LC, Hadwen WL (2022) Extreme events, loss, and grief—an evaluation of the evolving management of climate change threats on the Great Barrier Reef. Ecol Soc 27(1). 10.5751/ES-12964-270137

[CR88] Waters Y, Thompson K, Wilson K, Dean A (2025) Beyond threats, we need more information about action – How individuals see themselves (or not) in complex social-ecological systems. J Environ Manag 379: 124788. 10.1016/j.jenvman.2025.124788. 10.1016/j.jenvman.2025.124788 40056575

[CR89] Waters YL, Wilson KA, Dean AJ (2024) The role of iconic places, collective efficacy, and negative emotions in climate change communication. Environ Sci Policy, 151. 10.1016/j.envsci.2023.103635

[CR90] Whitmarsh L, Seyfang G, O’Neill S (2011) Public engagement with carbon and climate change: to what extent is the public ‘carbon capable’? Glob Environ Change 21(1):56–65. 10.1016/j.gloenvcha.2010.07.011.

[CR91] Wu J(Snow), Font X, Liu J (2021) Tourists’ pro-environmental behaviors: moral obligation or disengagement? J Travel Res 60(4):735–748. 10.1177/0047287520910787.

[CR92] Wynveen CJ, Sutton SG (2015) Engaging the public in climate change-related pro-environmental behaviors to protect coral reefs: the role of public trust in the management agency. Mar Policy 53:131–140. 10.1016/j.marpol.2014.10.030.

