# Supplementary material for: Stewardship Behaviour Among Residents of the Great Barrier Reef Region and the Role of Self-Efficacy
Source: Environ Manage. 2026 Jun 5;76(6):208. doi: 10.1007/s00267-026-02525-x (PMC13241464; doi:10.1007/s00267-026-02525-x)
Supplement: Supplementary file 1 — Supplementary information [file 267_2026_2525_MOESM1_ESM.docx]

# Online Resource 1

**STEWARDSHIP BEHAVIOUR AMONG RESIDENTS OF THE GREAT BARRIER REEF REGION AND THE ROLE OF SELF-EFFICACY**

**Environmental Management**

Jane Dousset, Matthew I Curnock, Ju-Han Zoe Wang, Tracy Schultz, Angela J. Dean

**Corresponding author**

Jane Dousset
[jane.dousset@my.jcu.edu.au](mailto:jane.dousset@my.jcu.edu.au)

James Cook University, Townsville QLD 4811

Table 1: Regression results for analysis of Reef-protecting actions and self-efficacy

|  | **Performance of Reef-protecting actions** | | | | **Self-efficacy** | | | |
| --- | --- | --- | --- | --- | --- | --- | --- | --- |
|  | **Coefficient (b)** | **Lower CI** | **Upper CI** | **p** | **Coefficient (b)** | **Lower CI** | **Upper CI** | **p** |
| Management satisfaction | -0.067* | -0.128 | -0.006 | .0303 | -0.336* | -0.628 | -0.044 | .024 |
| Perceived Reef health | -0.061 | -0.142 | 0.019 | .136 | -0.225 | -0.580 | 0.130 | .215 |
| Perceived threat (climate change) | -0.094 | -0.232 | 0.044 | .181 | 0.210 | -0.168 | 0.588 | .277 |
| Perceived threat (rubbish) | 0.074 | -0.078 | 0.226 | .342 | -0.004 | -0.356 | 0.348 | .983 |
| Trust (scientists) | -0.063 | -0.128 | 0.002 | .056 | 0.306* | 0.031 | 0.581 | .029 |
| Age | -0.098** | -0.140 | -0.055 | <.001 | 0.137 | -0.072 | 0.346 | .199 |
| Gender (female) | 0.299* | 0.270 | 0.569 | .030 | 0.108 | -0.216 | 0.432 | .512 |
| Place identity | 0.194** | 0.140 | 0.248 | <.001 | 0.090 | -0.181 | 0.363 | .512 |
| Reef-related income | 0.367** | 0.227 | 0.507 | <.001 | -0.004 | -0.527 | 0.519 | .988 |
| Hope | -0.057 | -0.132 | 0.018 | .134 | 0.019 | -0.326 | 0.364 | .916 |
| Moral obligation | 0.073* | 0.001 | 0.145 | .047 | 0.332* | 0.042 | 0.622 | .0251 |
| Normative belief | -0.021 | -0.083 | 0.040 | .495 | -0.062 | -0.237 | 0.113 | .488 |
| Pride | 0.005 | -0.070 | 0.080 | .899 | 0.165 | -0.141 | 0.471 | .292 |
| Sadness | -0.022 | -0.084 | 0.039 | .478 | -0.630** | -0.923 | -0.337 | <.001 |
| Self-efficacy | 0.129** | 0.081 | 0.177 | <.001 |  |  |  |  |
| Value (existence) | -0.010 | -0.097 | 0.076 | .817 | -0.137 | -0.456 | 0.182 | .398 |
| Education | 0.105* | 0.031 | 0.179 | .005 | 0.061 | -0.192 | 0.313 | .638 |
| Income | -0.016 | -0.093 | 0.060 | .676 | 0.192 | -0.046 | 0.430 | .115 |
| Knowledge | 0.324** | 0.253 | 0.395 | <.001 | 0.055 | 0.360 | 0.740 | .570 |

*Note.* CI = 95% confidence interval, LL = lower limit, UL = upper limit. * = p<.05, ** = p<.001
