# Supplementary material for: Stewardship Behaviour Among Residents of the Great Barrier Reef Region and the Role of Self-Efficacy
Source: Environ Manage. 2026 Jun 5;76(6):208. doi: 10.1007/s00267-026-02525-x (PMC13241464; doi:10.1007/s00267-026-02525-x)
Supplement: Supplementary file 2 — Supplementary information [file 267_2026_2525_MOESM2_ESM.docx]

**Online Resource Caption**

**Online Resource 1 –** This supplementary material shows the results of all regression analyses performed for this research paper in tabular format. It indicates the coefficient, lower and upper 95% confidence interval, and p-value for each explanatory factor in each regression analysis.
